# Are there socioeconomic inequalities in polypharmacy among older people? A systematic review and meta-analysis

**DOI:** 10.1186/s12877-023-03835-z

**Published:** 2023-03-18

**Authors:** Anum Iqbal, Charlotte Richardson, Zain Iqbal, Hannah O’Keefe, Barbara Hanratty, Fiona E. Matthews, Adam Todd

**Affiliations:** 1 School of Pharmacy, Population Health Sciences Institute, Newcastle University, King George VI Building, King’s Road, Newcastle Upon Tyne, NE1 7RU England; 2 School of Pharmacy, Newcastle University, Newcastle Upon Tyne, England; 3 Novartis International, Basel, Switzerland; 4 Population Health Sciences Institute, Newcastle University, Newcastle Upon Tyne, England; 5 Faculty of Medical Sciences, Population Health Sciences Institute, Newcastle University, Newcastle Upon Tyne, England

**Keywords:** Medication Usage, Socioeconomic Status, Health Inequalities, Polypharmacy, Ageing, Meta-analysis

## Abstract

**Background:**

Socioeconomic status (SES) may influence prescribing, concordance and adherence to medication regimens. This review set out to investigate the association between polypharmacy and an individual’s socioeconomic status.

**Methods:**

A systematic review and meta-analyses of observational studies was conducted across four databases. Older people (≥ 55 years) from any healthcare setting and residing location were included. The search was conducted across four databases: Medline (OVID), Web of Science, Embase (OVID) and CINAHL. Observational studies from 1990 that reported polypharmacy according to SES were included. A random-effects model was undertaken comparing those with polypharmacy (≥ 5 medication usage) with no polypharmacy. Unadjusted odds ratios (ORs), 95% confidence intervals (CIs) and standard errors (SE) were calculated for each study.

**Results:**

Fifty-four articles from 13,412 hits screened met the inclusion criteria. The measure of SES used were education (50 studies), income (18 studies), wealth (6 studies), occupation (4 studies), employment (7 studies), social class (5 studies), SES categories (2 studies) and deprivation (1 study). Thirteen studies were excluded from the meta-analysis. Lower SES was associated with higher polypharmacy usage: individuals of lower educational backgrounds displayed 21% higher odds to be in receipt of polypharmacy when compared to those of higher education backgrounds. Similar findings were shown for occupation, income, social class, and socioeconomic categories.

**Conclusions:**

There are socioeconomic inequalities in polypharmacy among older people, with people of lower SES significantly having higher odds of polypharmacy. Future work could examine the reasons for these inequalities and explore the interplay between polypharmacy and multimorbidity.

**Supplementary Information:**

The online version contains supplementary material available at 10.1186/s12877-023-03835-z.

## Introduction

The burgeoning impact of polypharmacy, often defined as the use of five or more medications [1], has become an increasing challenge for healthcare professionals. With the growing usage of medication, the term hyper/excessive polypharmacy has also been used, which refers to people typically using ≥ 10 medication at any one time [2]. The increased use of multiple medications has raised some concerns, particularly across older people, as this population is more likely to develop adverse drug events, including drug-drug interactions, non-adherence and falls [3, 4].

Increasing numbers of people are experiencing polypharmacy and this challenge has become a global public health concern. In the United Kingdom (UK), for example, the number of people experiencing polypharmacy has quadrupled over a 20-year period [5], while an Australian based study [6] highlighted a 52% increase in polypharmacy between 2006–2017.

In some contexts, the increasing trend of prescribing medication and the resulting polypharmacy is appropriate and necessary; multiple medications are often required to manage long-term conditions. As such, with rising multimorbidity and increasing life expectancy, polypharmacy may be clinically appropriate and thus reflective of treatment needs [7, 8]. However, there are situations where polypharmacy may be inappropriate and problematic; it is only possible to evaluate medication appropriateness by looking at individual patient preferences, circumstances, and contexts [9].

While previous studies have shown that certain patient-based factors, such as age, are associated with increased levels of polypharmacy, the role of socioeconomic factors such as education, income and occupation, is less clear. The literature suggests that such socioeconomic factors could play an important role in polypharmacy, with studies highlighting factors such as income [10–14] and employment [15–17] as possible contributors to the prevalence of polypharmacy. For example, a Swedish study [18] investigated the relationship between polypharmacy, socioeconomic status (SES) and inappropriate medication usage. The results showed that lower levels of education were associated with increased levels of polypharmacy and potential drug-drug interactions. Further to this, some authors have highlighted the rising concerns of low SES on the adverse impact on life expectancy, access to healthcare and multimorbidity. One study [19] has showed that low SES was associated with 2.1-year reduction in life expectancy for men and women aged 40–85 years. Such an impact is important given there is potential that those of lower SES being exposed to higher levels of polypharmacy and thus the associated harms.

Whilst there is significant academic interest on this topic, there is no single review and meta-analysis which draws together the current literature around polypharmacy and how prevalence may differ according to SES. Therefore, this systematic review and meta-analysis aimed to investigate the association between socioeconomic status and the prevalence of polypharmacy, in older people.

## Methods

This review was registered with accordance to The International Prospective Register of Systematic Reviews PROSPERO (CRD42021285455) and reported according to Preferred Reporting Items for Systematic Reviews and Meta-Analyses (PRISMA).

### Data sources

A literature search was conducted across the following databases: Medline (OVID), Web of Science, Embase (OVID) and CINAHL, from inception to July 2021. The search was developed around three key terms: ‘polypharmacy’ ‘socioeconomic status’ and ‘ageing’ which captured the literature surrounding the key purpose of this review. The full search strategy can be found in Supplementary 1. Additional articles were identified through hand searching reference lists and forward citations of eligible articles.

### Study selection

Studies included in this review met the following criteria:

- Population: older people. In line with previous reviews [20] older people were defined as people aged ≥ 55 years. Studies required at least 50% of participants to be over ≥ 55 years.
- Exposure: lower socioeconomic status
- Comparison: higher socioeconomic status
- Outcome: receipt of polypharmacy
- Setting: all settings were considered, irrespective of country, private or public healthcare systems.
- Study type: all observational study types including cohort and cross sectional.

To be eligible for inclusion, articles were required to be available as full text and published in English. After discussion with the review team, prescribing practices before 1990 were considered to be less relevant to address the review question, and hence articles published before 1990 were not eligible for inclusion.

### Selection criteria and screening

Records were uploaded to End Note and duplicates were removed. Rayyan QCRI was used for screening of titles and abstracts, which was conducted by one reviewer (AI). Two reviewers (CR,ZI) independently reviewed 25% of extracted articles. Full text screening was conducted by one reviewer (AI) and checked by another reviewer (CR,ZI); any discrepancies were resolved through discussion and consensus (AT). The level of agreement between the review team was determined by a Kappa score – 0.85 showing excellent agreement.

### Data extraction and quality appraisal

The following information was extracted using a prepopulated data extraction form: first author, year, study data, participant characteristics, socioeconomic measure, main data extraction in relation to SES and polypharmacy. Data extraction was conducted by one reviewer (AI) and checked by the review team (CR,ZI); any discrepancies were resolved through discussion and consensus (AT). For the quality appraisal, one reviewer (AI) used the relevant critical appraisal tool from the Joanna Briggs Institute (JBI), which was checked (CR,ZI); any discrepancies were resolved through discussion and consensus (AT).

### Statistical methods

Random effects meta-analysis was performed to assess the association between a given socioeconomic factor and polypharmacy. Eligibility criteria for studies to be included in the meta-analysis were as follows: i) unadjusted raw data reporting polypharmacy rates for an individual socioeconomic factor ii) total participants/information to identify total number of participants displaying polypharmacy and no polypharmacy for the socioeconomic factor being investigated. Unadjusted odds ratios, confidence intervals and standard errors were then calculated independently by two reviewers (AI, FM). I^2^ was calculated to determine the degree of heterogeneity amongst the studies. Odd ratios were calculated by comparing the bottom 25% of each study population to the remainder participants for each given socio-economic factor. Log odd ratios and SE were then entered into Revman 5.4 to generate forest plots.

## Results

### Literature search

**Fig. 1:**
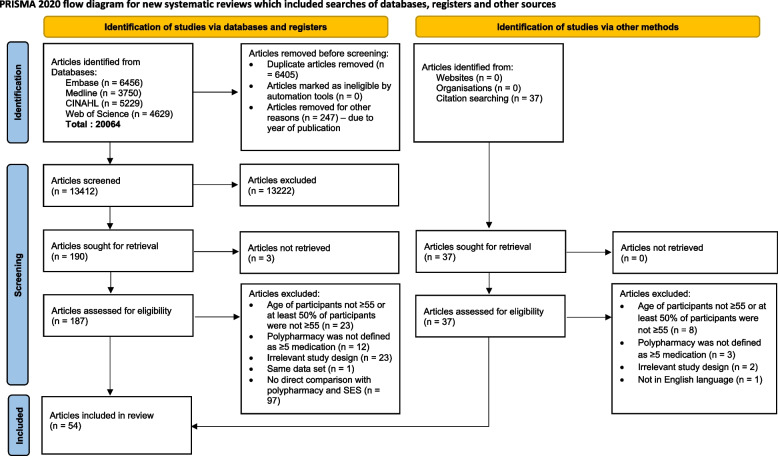
Study selection and exclusion according to the PRISMA flow diagram

Searches retrieved 20,064 citations. After de-duplication, 13,412 articles were screened for eligibility based on title and abstract. A further 187 articles were progressed to full text screening, which resulted in 54 articles meeting the inclusion criteria (Fig. 1); 13 articles were excluded from the meta-analysis.

### Study characteristics

The 54 included studies used a range of measures to assess SES factors. Fifty studies focussed on education [16, 18, 20–67], 18 studies on income [16, 23, 30, 32–34, 37, 41, 46–48, 50, 53, 61, 63, 64, 66, 68], 6 on wealth [22, 27, 45, 57, 67, 69], 4 studies on occupation [23, 44, 48, 57], 7 studies on employment [16, 17, 49, 58, 60, 63, 66], 5 studies on social class [17, 25, 36, 38, 60], 2 used SES categories [31, 70] and 1 used area-level deprivation [20]. Studies were conducted across a range of countries as follow: India [60, 66], Jordan [21], Netherlands [70], Sweden [18, 23, 39, 52], Spain [25], Belgium [26, 30], Pakistan [29, 68], UK [17, 20, 36, 38, 69], China [33, 44, 59, 65], Japan [46, 67], Singapore [40], Kuwait [42], Malaysia [43, 58], Poland [45], Togo [47], Saudi Arabia [55, 61], Taiwan [56], Vietnman [57]. Most studies were conducted within Brazil [28, 31, 32, 37, 41, 48, 50, 51, 53, 62, 63] and the US [16, 24, 27, 34, 35, 49, 54, 64]. One study included participants from across Europe and Israel [22]. Studies ranged in size from 59 [24] to 1,742,336 [52] participants. Full study characteristics can be found in Supplementary 2.

### Quality appraisal

The included 54 studies scored in the range 6–8, out of a possible 8 (S2). Articles often scored poorly on identifying and reporting confounders. The majority scored well on displaying inclusion criteria, using appropriate statistical analysis, and describing subjects and setting.

### Association between education and polypharmacy

**Fig. 2:**
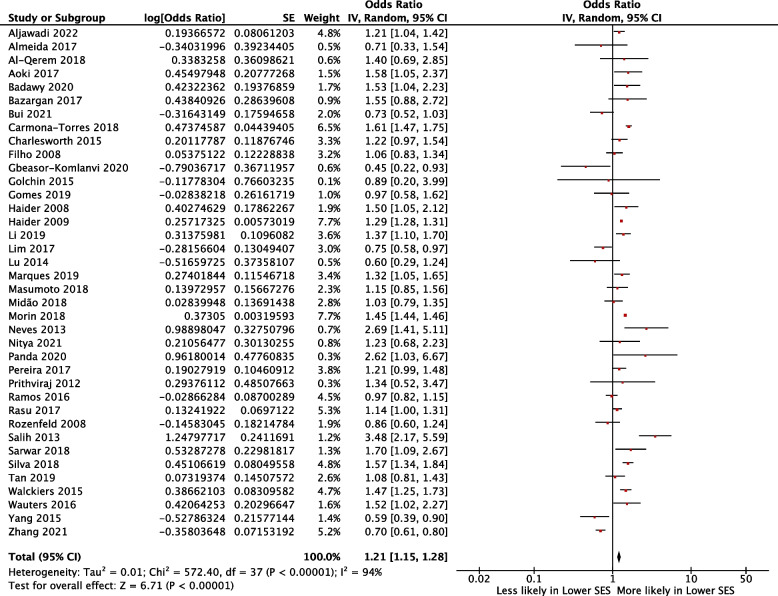
Forest plot showing the likelihood of polypharmacy according to education

Fifty studies [16, 18, 20–67] investigated the association between education and polypharmacy, 38 studies [18, 21–31, 33, 35, 37, 40–42, 44, 46–48, 50–55, 57–61, 63–67] were eligible for meta-analysis giving a pooled OR of 1.21 (95% CI 1.15–1.28; I^2^ = 94%) for receipt of polypharmacy in those of lower education when compared to higher education (Fig. 2).

### Association between income and polypharmacy

**Fig. 3:**
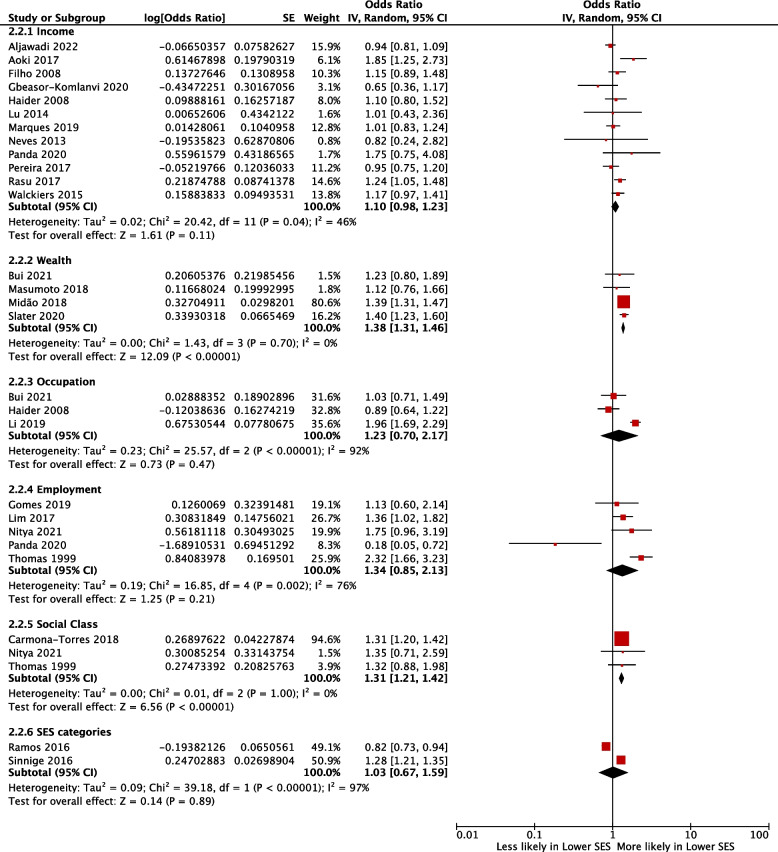
Forest plot showing the likelihood of polypharmacy according to income, wealth, occupation, employment, social class and SES categories

Eighteen studies [16, 23, 30, 32–34, 37, 41, 46–48, 50, 53, 61, 63, 64, 66, 68] investigated the association between income and polypharmacy, 12 studies [23, 30, 33, 37, 41, 46, 47, 50, 53, 61, 64, 66] were eligible for meta-analysis giving a pooled OR of 1.10 (95% CI 0.98–1.23; I^2^ = 46%) for receipt of polypharmacy in those of a low compared to high income (Fig. 3).

### Association between wealth and polypharmacy

Six studies [22, 27, 45, 57, 67, 69] investigated the association between wealth and polypharmacy, 4 studies [22, 57, 67, 69] were eligible for meta-analysis, giving a pooled OR of 1.38 (95% CI 1.31–1.46; I^2^ = 0%) for receipt of polypharmacy in those of less wealthier backgrounds (Fig. 3).

### Association between occupation, employment, and polypharmacy

Four studies [23, 44, 48, 57] reported the association between occupation and polypharmacy, and 7 [16, 17, 49, 58, 60, 63, 66] reported the association between employment and polypharmacy. Three studies [23, 44, 57] assessing occupation and 5 studies [17, 58, 60, 63, 66] assessing employment were eligible for meta-analysis (Fig. 3). A pooled OR of 1.23 (95% CI 0.70 - 2.17; I^2^ = 92%) was calculated for those in receipt of polypharmacy from lower occupations. Similarly, the pooled OR was 1.34 (95% CI 0.85–2.13; I^2^ = 76%) for receipt of polypharmacy in unemployed, compared to employed, individuals.

### Association between social class, SES and polypharmacy

Five studies [17, 25, 36, 38, 60] reported the association between social class and polypharmacy, and 2 studies [31, 70] focused on SES and polypharmacy. Three studies [17, 25, 60] assessing social class were eligible for meta-analysis and the pooled OR was 1.31 (95% CI 1.21–1.42; I^2^ = 0%) for receipt of polypharmacy in those of lower social class compared to higher social class. Two studies [31, 70] assessing SES were eligible for meta-analysis and the pooled OR was 1.03 (95% CI 0.67–1.59; I^2^ = 97%) for receipt of polypharmacy for those of lower, compared to higher, SES.

## Discussion

### Main finding

This systematic review and meta-analysis found that, overall, polypharmacy is associated with lower socioeconomic status. In particular, pooled estimates revealed a significant association when using education as a marker of SES: those of lower educational backgrounds had 21% higher odds to be in receipt of polypharmacy when compared to those of higher education. Significant associations were also observed when wealth and social class were used as SES measures. Similar trends were observed for income, occupation, employment and SES categories, although the results did not reach statistical significance. The majority of the studies included in this review used education as a marker of socioeconomic status, while fewer studies used socioeconomic measures such as occupation, income and social class.

### Comparison with other reviews

To the best of our knowledge this is the first systematic review and meta-analysis that has been carried out exploring the relationship between polypharmacy and socioeconomic status; focusing on an ageing population irrespective of co-morbidities or drug class. A previous review revealed that there were significant associations between socioeconomic factors, such as education and deprivation, and multimorbidity whereby people of lower socioeconomic status have a higher risk of multimorbidity [71]. This work, unlike our review, did not focus on polypharmacy or older people, but can be used as a possible justification of our findings. Given, older people with multimorbidity are more likely to use more medications, and people of lower SES have higher risk of developing multimorbidity, may help explain – at least in part – some of our findings. However, the interplay between multimorbidity and polypharmacy is likely to be complex and should be the subject of further investigation. For example, those of higher SES may still have high levels of multimorbidity but their social status has the potential influence to ensure that they can better manage their conditions, have access to better healthcare services, reduced waiting time to see healthcare professionals, all factors of which have the potential to influence medication usage. Some of the literature has touched on the aspect of healthcare access [72] and the so called ‘wealth health’ gradient, showcasing that socioeconomic status has a direct influence on healthy ageing. Others have also revealed the potential influence that patients have on the medication that is prescribed to them [73, 74]. Those of higher SES are often better at navigating healthcare systems (both public and private) and are potentially more able to obtain multiple clinical opinions for their concerns resulting in an increased likelihood of being prescribed the medications they want or believe they need. Prosser et al. [75] showcases prescribing of medication is often patient mediated, and thus more costly, beneficial treatment may be prescribed to those that are more proactive in their health, often those of higher SES.

Other reviews that have been conducted which investigate treatment adherence and subsequent factors have all revealed that socioeconomic status plays an integral role [76–79]. Whilst these reviews are not primarily focused on polypharmacy or the ageing population, they provide important information on medication usage: treatment adherence. The reviews have shown statistical significance whereby socioeconomic factors, such as lower income, unemployment, and lower education, are associated with medication non-adherence. It can therefore be suggested that non-adherence to medication could play an important role in deteriorating health and has a subsequent effect in the rise of multimorbidity and polypharmacy. It is also worth noting that, although we calculated unadjusted odds ratios for the meta-analysis, many of the studies included in this review did adjust for multimorbidity in their analysis and still yielded statistical significant results—with a higher odds of polypharmacy in older people of lower socioeconomic status.

### Individual socioeconomic factor results

To conceptualise socioeconomic status, this systematic review included studies employing different methods to assess socioeconomic status, including education, income, and employment.

With respect to education status, the overall findings of the review revealed that older people with lower educational backgrounds are of greater odds of polypharmacy. The literature suggests there are several reasons as to why this may be the case. Firstly, individuals with lower levels of education can be viewed as playing a less proactive role in preventive measures to improve/maintain their health, and thus are of greater risks of developing conditions that would likely result in them taking multiple medications [80, 81]. Secondly, some have suggested those of lower education are less likely to challenge healthcare professionals and be less involved with shared decision making [82, 83]. This, therefore, may have the potential for people to take additional medication without requiring a detailed explanation from their healthcare professional [84, 85]. Such patients are also seen to be less concerned in asking key questions regarding their medical care [86], thus it can be questioned whether they are truly aware of the potential additional harm that may be associated with taking multiple medications. However, other researchers have argued that people with lower levels of education may be less likely to approach healthcare professionals for medication and thus inevitably display lower levels of medication usage [87].

Previous work has shown that people entitled to free medications were more likely to display higher levels of polypharmacy [88]. In most instances, unemployed individuals, or individuals with lower income would be entitled to free prescription coverage and as there is no direct cost to the patient, they would be more likely to show higher medication usage. These findings can be used to support our results when assessing employment or income as a marker of socioeconomic status—that is unemployment or low income is associated with more polypharmacy. Out of pocket cost of medication, has a clear influence on the likelihood of individuals not wanting to take more medication. However, this can also be influenced by education attainment, and often people with higher income are more likely to have higher education attainment. As previously discussed, people with higher education attainment maybe more proactive in making decisions about their health and also be aware of the risks associated with polypharmacy.

### Strengths and limitations

This systematic review and meta-analysis showcased comprehensive findings in relation to the association of socioeconomic status on polypharmacy in older people. Whilst our approach was comprehensive and the methodology robust, we do acknowledge that our work has limitations. Firstly, the definition we used to conceptualise older people (≥ 55 years) was arbitrary – the appropriateness of which can be debated. It is important to acknowledge that our approach was in keeping with previous reviews in the field of older people and polypharmacy. For example, the work of Davies et al. was used to help establish our definition of older people; our initial scoping searches also supported using the ≥ 55 years definition, as this conservative approach enabled the inclusion of key literature, and ensured that articles were not excluded for being too broad in their inclusion criteria. Another advantage to this review was that included studies were from a variety of low, middle, and high-income countries. Whilst this is advantageous, it is important to acknowledge that studies undertaken in a variety of healthcare systems have been included, this also contributes to the large heterogeneity observed. In some cases, it was challenging to ascertain how studies assessed different socioeconomic factors; for example, the definition of ‘high’ education varied across studies. To account for these variations and differences, when conducting the meta-analysis, the decision was made to compare the lowest 25% of each population (in terms of SES factor) to the remainder of the population. This approach also ensured that all participants within the included studies were included and factored into the meta-analysis.

### Future work

Whilst this review highlights that there are socioeconomic inequalities in polypharmacy – whereby people of lower SES are more likely to receive polypharmacy, the work does not explore the potential causes of this. It would be useful to understand how people of lower socioeconomic status engage with medication reviews, with such reviews having the potential to aid deprescribing decisions and possibly reduce polypharmacy. Previous work has shown that certain populations (e.g. ethnic minority communities), struggle to engage in medication review services [89, 90]. Whilst this review has demonstrated that there is socioeconomic inequalities in polypharmacy, it is important that policies be put in place to enable healthcare professionals to work towards reducing such inequalities and not exacerbate them further. At present, certain patient demographic (e.g. age) or medication-related factors (e.g. using a high risk medication) may trigger a medication review. Our work suggests that other factors, such as SES, could be used to trigger for medication related review services. Indeed, health inequalities have been at the forefront of healthcare policy formulation for many years, particularly in the UK, especially since the wide-spread appreciation of the existence of a ‘postcode lottery’ [91]. This concept suggests that healthcare standards and subsequently polypharmacy and medication utilisation can be influenced by an individual’s geographic location. For example, people living in the North of England are more likely to use an opioid analgesic, compared to people living the South of England [92]. If factors such as education/poor healthcare literacy play a critical role in polypharmacy it is important for healthcare professionals to understand the needs of their patients and factor these into consultations.

## Conclusion and implications

There are significant socioeconomic inequalities in polypharmacy among older people, whereby people with lower SES have higher odds of being in receipt of polypharmacy. This association was found using a range of markers of SES including education, and social class. Future work could examine the reasons for these inequalities and explore the interplay between polypharmacy and multimorbidity.

## Supplementary Information

**Additional file 1.** 

**Additional file 2.** 

## Data Availability

All data generated or analysed during this study are included in this published article [and its supplementary information files].
