# Supplementary material for: Are there socioeconomic inequalities in polypharmacy among older people? A systematic review and meta-analysis
Source: BMC Geriatr. 2023 Mar 18;23:149. doi: 10.1186/s12877-023-03835-z (PMC10024437; doi:10.1186/s12877-023-03835-z)
Supplement: Supplementary file 1 — Additional file 1. [file 12877_2023_3835_MOESM1_ESM.docx]

**Search Strategies**

**Embase (OVID)**

| 1. | exp Socioeconomic Factors/ or socioeconomic*.mp. |
| --- | --- |
| 2. | social class.mp. |
| 3. | *social class/ or *social factors/ or *sociology, medical/ |
| 4. | socioeconomic factor*.mp. |
| 5. | socioeconomic statu*.mp. |
| 6. | income.mp. or *Income/ |
| 7. | (income adj6 category).mp. |
| 8. | household income.mp. |
| 9. | employment status.mp. |
| 10. | Unemploy*.mp. |
| 11. | Employment characteristic*.mp. |
| 12. | ((Education or Employ* or Unemploy*) adj6 (demographic or variable)).mp. |
| 13. | education.mp. |
| 14. | Education level.mp. |
| 15. | index of multiple deprivation.mp. |
| 16. | socioeconomic position.mp. |
| 17. | Carstairs index.mp. |
| 18. | townsend index.mp. |
| 19. | area level deprivation.mp. |
| 20. | wealth.mp. |
| 21. | occupation*.mp. or *Occupations/ |
| 22. | poverty level.mp. |
| 23. | exp polypharmacy/ |
| 24. | polypharmacy.mp. |
| 25. | hyperpolypharmacy.mp. |
| 26. | medic* usage.mp. |
| 27. | medic* utili*.mp. |
| 28. | ((number or multiple or more or total or prescrib* or many or several) adj2 (medicine* or medication* or drug*)).mp. |
| 29. | polypharmacotherapy.mp. |
| 30. | polymedic*.mp. |
| 31. | polydrug*.mp. |
| 32. | polytherapy.mp. |
| 33. | multimedic*.mp. |
| 34. | exp "Aged, 80 and over"/ or exp Aged/ |
| 35. | exp Retirement/ |
| 36. | exp Veterans/ |
| 37. | Geriatrics/ |
| 38. | exp Residential Facilities/ |
| 39. | exp Nursing Homes/ |
| 40. | ((residential or nursing or care) adj home*).mp. |
| 41. | exp Long-Term Care/ |
| 42. | ((old* or elder*) adj2 (adult or person* or people* or age*)).mp. |
| 43. | "aged, over 55".mp. |
| 44. | *Poverty/ or poverty.mp. |
| 45. | 1 or 2 or 3 or 4 or 5 or 6 or 7 or 8 or 9 or 10 or 11 or 12 or 13 or 14 or 15 or 16 or 17 or 18 or 19 or 20 or 21 or 22 or 44 |
| 46. | 34 or 35 or 36 or 37 or 38 or 39 or 40 or 41 or 42 or 43 |
| 47. | 22 or 23 or 24 or 25 or 26 or 27 or 28 or 29 or 30 or 31 or 32 or 33 |
| 48. | 45 and 46 and 47 |

**Medline (OVID)**

| 1. | exp Socioeconomic Factors/ or socioeconomic*.mp. |
| --- | --- |
| 2. | social class.mp. |
| 3. | *social class/ or *social factors/ or *sociology, medical/ |
| 4. | socioeconomic factor*.mp. |
| 5. | socioeconomic statu*.mp. |
| 6. | income.mp. or *Income/ |
| 7. | (income adj6 category).mp. |
| 8. | household income.mp. |
| 9. | employment status.mp. |
| 10. | Unemploy*.mp. |
| 11. | Employment characteristic*.mp. |
| 12. | ((Education or Employ* or Unemploy*) adj6 (demographic or variable)).mp. |
| 13. | education.mp. |
| 14. | Education level.mp. |
| 15. | index of multiple deprivation.mp. |
| 16. | socioeconomic position.mp. |
| 17. | Carstairs index.mp. |
| 18. | townsend index.mp. |
| 19. | area level deprivation.mp. |
| 20. | socioeconomic*.mp. or exp socioeconomics/ |
| 21. | wealth.mp. |
| 22. | occupation*.mp. or *Occupations/ |
| 23. | poverty level.mp. |
| 24. | poverty.mp. |
| 25. | exp polypharmacy/ |
| 26. | polypharmacy.mp. |
| 27. | hyperpolypharmacy.mp. |
| 28. | medic* usage.mp. |
| 29. | medic* utili*.mp. |
| 30. | ((number or multiple or more or total or prescrib* or many or several) adj2 (medicine* or medication* or drug*)).mp. |
| 31. | polypharmacotherapy.mp. |
| 32. | polymedic*.mp. |
| 33. | polydrug*.mp. |
| 34. | polytherapy.mp. |
| 35. | multimedic*.mp. |
| 36. | exp "Aged, 80 and over"/ or exp Aged/ |
| 37. | exp Retirement/ |
| 38. | exp Veterans/ |
| 39. | Geriatrics/ |
| 40. | exp Residential Facilities/ |
| 41. | exp Nursing Homes/ |
| 42. | ((residential or nursing or care) adj home*).mp. |
| 43. | exp Long-Term Care/ |
| 44. | ((old* or elder*) adj2 (adult or person* or people* or age*)).mp. |
| 45. | "aged, over 55".mp. |

**CINAHL**

| S1 | TX socioeconomic* OR "social class*" OR poverty OR wealth OR "socioeconomic factor*" OR "socioeconomic statu*" OR income OR "household income" OR "employment status" OR unemploy* OR "employment characteristics" OR occupation* OR education OR "education level" OR "index of multiple deprivation" OR "socioeconomic position" OR "Carstairs index" OR "townsend index" OR "area level deprivation" OR "poverty level" OR poverty |
| --- | --- |
| S2 | TX income N6 category |
| S3 | TX ((Education or Employ* or Unemploy*) N6 (demographic or variable)) |
| S4 | TX ((number or multiple or more or total or prescrib* or many or several) N2 (medicine* or medication* or drug*)) |
| S5 | TX polypharmacy OR hyperpolypharmacy OR "medic* usage" OR "medic* utili*" OR polypharmacotherapy OR polymedic* OR polydrug* OR polytherapy OR multimedic*) |
| S6 | TX "aged, over 80" OR retirement OR veteran OR geriatric* OR "residential home" OR "nursing home" OR "long term care" OR aged OR "age, over 55" |
| S7 | TX (residential or nursing or care) N home*) |
| S8 | S1 OR S2 OR S3 |
| S9 | S4 OR S5 |
| S10 | S6 OR S7 |
| S11 | S8 AND S9 AND S10 |

**Web of Science**

1. TS=(socioeconomic* OR "social class*" OR poverty OR wealth OR "socioeconomic factor*" OR "socioeconomic statu*" OR income OR "household income" OR "employment status" OR unemploy* OR "employment characteristics" OR occupation* OR education OR "education level" OR "index of multiple deprivation" OR "socioeconomic position" OR "Carstairs index" OR "townsend index" OR "area level deprivation" OR "poverty level" OR poverty )
2. TS=((Education or Employ* or Unemploy*) NEAR/6 (demographic or variable))
3. TS=(polypharmacy OR hyperpolypharmacy OR "medic* usage" OR "medic* utili*" OR polypharmacotherapy OR polymedic* OR polydrug* OR polytherapy OR multimedic*)
4. TS=((number or multiple or more or total or prescrib* or many or several) NEAR/2 (medicine* or medication* or drug*))
5. TS=(income NEAR/6 category)
6. TS=("aged, over 80" OR retirement OR veteran OR geriatric* OR "residential home" OR "nursing home" OR "long term care" OR aged OR "age, over 55")
7. TS=((residential or nursing or care) NEAR home*)
8. #1 OR #2 OR #5
9. #3 OR #4
10. #6 OR #7
11. #8 AND #9 AND #10
