# Supplementary material for: Are there socioeconomic inequalities in polypharmacy among older people? A systematic review and meta-analysis
Source: BMC Geriatr. 2023 Mar 18;23:149. doi: 10.1186/s12877-023-03835-z (PMC10024437; doi:10.1186/s12877-023-03835-z)
Supplement: Supplementary file 2 — Additional file 2. [file 12877_2023_3835_MOESM2_ESM.docx]

| First Author, year | Country | Study Data | Participant characteristics | Socioeconomic Measure | Main Data Extraction in relation to SES and Polypharmacy | Quality Appraisal |
| --- | --- | --- | --- | --- | --- | --- |
| Al-Qerem W *et al* (2018) | Jordan | Prescriptions obtained from pharmacies and hospitals in Amman, Madaba and Zarqa (Jordan) : October 2017 - January 2018 | - Total 367 - Age ≥60 | Education | Education: OR 1.40 SE 0.36 | 6/8 |
| Midão L *et al* (2018) | Austria, Belgium, Croatia, the Czech Republic, Denmark, Estonia, France, Germany, Greece, Italy, Luxembourg, Poland, Portugal, Spain, Sweden, Switzerland and Slovenia) and Israel | SHARE – Survey of Health, Ageing and Retirement in Europe | - Total 29,342 - Age ≥65 | Education  Wealth – shortage of money | Wealth/Shortage of money: OR 1.39 SE = 0.03  Education  OR 1.03 SE = 0.14 | 8/8 |
| Panda M *et*  al  (2020) | India | Primary Data – collected from rural health and training centres: December 2018 – July 2019 | - Total 300 *analysis based on N=127* - Age ≥60 | Education  Income  Employment | Education – OR 2.62 SE 0.48  Income – OR 1.75 SE 0.43  Employment – OR 0.18 SE 0.69 | 8/8 |
| Sinnige J *et al* (2016) | Netherlands | Electronic Medical Records through NIVEL Primary Care Database (NIVEL-PCD) | - Total 45,731 *analysis based on N=45,496* - Age ≥55 | *‘SES Category’ combining*  Income  Employment  Education | SES OR 1.28 SE 0.03 | 8/8 |
| Haider I, *et al*  (2008) | Sweden | The SWEOLD (Swedish Panel Study of Living Conditions of the Oldest Old) | - Total 621 - Age ≥77 | Education  Occupation  Income | Education OR 1.50 SE 0.18  Occupation – OR 0.89 SE 0.16  Income – OR 1.10 SE 0.16 | 8/8 |
| Golchin N *et al* (2015) | United States | Survey – under the Cuyahoga Country Board of Health | - Total 59 - Age ≥65 | Education | Education  OR 0.89 SE 0.77 | 6/8 |
| Carmona-Torres J *et al* (2018) | Spain | National Health Survey (2006, 2011 and 2012) and European Health Survey (2009 and 2014) | - Total 26,277 - Age ≥65 | Education  Social Class | Education – OR 1.61 SE 0.04  Social Class – OR 1.31 SE 0.04 | 7/8 |
| Wauters M *et al* (2016) | Belgium | BELFRAIL Study | - Total 503 - Age ≥80   *analysis based on N=473* | Education | Education – OR 1.52 SE 0.20 | 8/8 |
| Charlesworth C *et al* (2015) | United States | National Health and Nutrition Examination Survey (1988-2010) | - Total 13,869. *Data used for 2009-2010 N= 1,523* - Age ≥65 | Education  Wealth | Education  OR 1.22 SE 0.12  *Income poverty ratio taken from study analysis P 0.998* | 8/8 |
| Rozenfeld S *et al* (2008) | Brazil | Survey – Uso de medicamentos por aposentados brasileiros  (Medication use by Brazilian retirees) | - Total 577 - Age ≥60 | Education | Education  OR 0.86 SE 0.18 | 6/8 |
| Sarwar M *et al* (2018) | Pakistan | Primary data collection across various healthcare settings (tertiary care, private and public hospitals) between 1 December 2017 – 28 February 2018 | - Total 385 - Age ≥65 | Education | Education OR 1.70 SE 0.23 | 6/8 |
| Walckiers D *et al* (2015) | Belgium | Belgian Health Interview Survey (2008) | - Total 2835 - Age ≥65 | Education  Income | Education  OR 1.47 SE 0.08  Income – OR 1.17 SE 0.10 | 8/8 |
| Thomas H *et al* (1999) | Wales | Survey – The Caerphilly Prospective Study | - Total 1906 - Age ≥56-75 | Employment  Social Class | Social class –  OR 1.32 SE 0.21  Employment -  OR 2.32 SE 0.17 | 6/8 |
| Ramos L *et al* (2016) | Brazil | Survey - Access, Use and Promotion of Rational Use of Medicines (PNAUM) | - Total 6844 - Age ≥60 | SES  Education | SES -  OR 0.82 SE 0.07  Education:  OR 0.97 SE 0.09 | 8/8 |
| Carvalho M *et al* (2012) | Brazil | Primary data collection - S*aúde, Bem-Estar e Envelhecimento :* SABE Health, Wellbeing and Ageing study | - Total 2143 - Age ≥65 | Income  Education | *Taken from data table in paper*  Income:  Tercile 1: 1.0  Tercile 2: 1.5 (1.0-2.2) *P*  0.057  Tercile 3: 1.3 (0.9-1.9) P 0.090  Education  Year 0: 1.0 Year 1 to 3: 0.8 (0.5 – 1.3) 0.339  Year 4 to 11 : 1.0 (0.6 – 1.7) 0.859  Year 12 to 22 1.4 (0.6 – 3.1) 0.380 | 8/8 |
| Lu J *et al* (2014) | China | Project of Longevity and Aging in Dujiangyan Study | - Total 859 - Age ≥90 | Income  Education | Income: OR 1.01 SE 0.43  Education OR 0.60 SE 0.37 | 7/8 |
| Assari S *et al* (2019) | United States | Primary data collection - Sacramento Area Latino Study on Aging (SALSA, 1996-2008) | - Total 632 - Age ≥65 | Income  Education  Employment | *Taken from data table in paper*  Education: OR 0.97 *P* 0.414  Income: OR 1.15 *P* 0.296  Employment OR 0.21 *P* 0.042 | 7/8 |
| Perry B *et al* (2001) | United States | Survey - National Health and Nutritional Examination Survey III 1988-1994 | - Total 5249 - Age ≥65 | Income  Education | *Taken from data table in paper*  *Beta weights*  Income: 0.07  Education: 0.28 | 7/8 |
| Prithviraj G *et al* (2012) | United States | Primary data – collected from patient from ambulatory oncology clinics at an academic centre (February 1^st^ 2008 - September 30^th^ 2009) | - Total 117 - Age ≥65 | Education | Education  OR 1.34 SE 0.49 | 7/8 |
| Rawle M *et al* (2018) | England, Scotland and Wales | Survey - MRC National Survey for Health and Development | - Total 2122 - Age ≥69 | Education  Social Class | *Taken from data table in paper*  Education  None (ref) *P* 0.010  Social class  Manual (ref) *P* 0.592 | 8/8 |
| Filho A *et al* (2008) | Brazil | Primary Data - Bambuí Project | - Total 1544 - Age ≥60 | Education  Income | Education –  OR 1.06 SE 0.12  Income  OR 1.15 SE 0.13 | 8/8 |
| Davies L *et al* (2021) | England | Primary Data – collected from general practices in Newcastle or North Tyneside: Newcastle 85+ study | - Total 845 - Age ≥85 | Area - level  Deprivation | *Taken from data table in paper*  (75th centile IMD) OR 1.04, 95% CI: 0.76– 1.42 and people living in socioeconomic disadvantage (<25th centile IMD) OR1.03, 95% CI: 0.76–1.39 | 7/8 |
| Chen Y *et al* (2001) | England and Wales | Survey - Cognitive Function and Ageing Study 1991- 1994 | - Total 12,489 - Age ≥65 | Social Class  Education | *Taken from data table in paper*  Social class (ref: class I & II)  ClassI&II 1.00 (0.90±1.12)  Class IIIa 1.21 (1.04±1.42)  Class IIIb 1.22 (1.12±1.34)  ClassIV&V 1.30 (1.15±1.47) Education (ref: ≥10 y)  ≤ 8 years 1.08 (0.88-1.31)  9 years 1.00 (0.93-1.07)  ≥ 10 years 1.00 (0.90-1.11) | 8/8 |
| Doheny M *et al* (2021) | Sweden | Primary data – collected from registered inhabitants  aged ≥65 31^st^ December 2014 | - Total 291,800 - Age ≥65 | Education | *Taken from data table in paper*  Education level:  Post-secondary OR 1.00  Primary OR 1.60  Secondary OR 1.28 | 8/8 |
| Slater N *et al* (2020) | England | The English Longitudinal Study of Ageing (ELSA) | - Total 7730 - Age ≥60 - 6035 | Wealth | Wealth –  OR 1.40 SE 0.07 | 8/8 |
| *Slater N *et al* (2018) | England | The English Longitudinal Study of Ageing (ELSA) | - Total 7730 - Age ≥60 - 6035 | Wealth | N/A | N/A |
| Masumoto S *et al* (2018) | Japan | Primary Data – collected from Japanese outpatient primary care clinics: January to March 2016 | - Total 740 - Age ≥65 | Wealth  Education | Wealth – OR 1.12 SE 0.20  Education – OR 1.15 SE 0.16 | 8/8 |
| Tan Y et al (2019) | Singapore | National sample – wave 3 of the Panel on Health and Ageing of Singaporean Elderly (PHASE) | - Total 1499 - Age ≥66 | Education | Education  OR 1.08 SE 0.15 | 8/8 |
| Neves S et al (2013) | Brazil | Primary data from those living in urban areas covered by the Family Health Strategy program in Recife | - Total 432 - Age ≥60 | Education  Income | Education  OR 2.69 SE 0.33  Income OR 0.82 SE 0.63 | 7/8 |
| Badawy N et al 2020 | Kuwait | Questionnaire based Survey March to July 2017 | - Total 500 - Age ≥65 | Education | Education  OR 1.53 SE 0.19 | 7/8 |
| Ong S et al (2018) | Malaysia | The National Medical Care Survey | - Total 22,832 - Age ≥65 | Education | *Taken from data table in paper*  Education – reference no formal education  Primary – OR 1.61 (1.12-2.32)  Secondary – OR 1.44 (0.93-2.23)  Tertiary – OR 1.08 (0.47-2.5) | 7/8 |
| Li J et al (2019) | China | Data from an existing computerised medical record database 2012-2017 | - Total 3370 ≥60 *n=2122* - Age ≥18 | Education  Occupation | Occupation  OR 1.96 SE 0.08  Education  OR 1.37 SE 0.11 | 7/8 |
| Neumann-Podczaska et al 2022 | Poland | PolSenior - nationwide multidisciplinary project conducted by International Institute of molecular Cell Biology | - Total 4793 - Age ≥65 years | Education  Self-reported poverty/Wealth | *Polypharmacy* 5+ vs 1-4  Education – OR 1.09 SE 0.08  Wealth – OR 0.95 SE 0.09 | 7/8 |
| Aoki T et al (2017) | Japan | Primary care practice-based research network October 2015-February 2016 | - Total 544 - Age ≥20 years , ≥61 *n=*454 | Education  Income | Education OR 1.58 SE 0.21  Income OR 1.85 SE 0.20 | 8/8 |
| Gbeasor-Komlanvi (2020) | Togo | Interview and questionnaires March to June 2017 | - Total 370 - ≥60 years | Education  Income | Education OR 0.45 SE 0.37  Income OR 0.65 SE 0.30 | 7/8 |
| Sarwar M et al (2017) | Pakistan | Data collected from December 2015 to March 2016 Tertiary Care Hospitals Punjab | - Total 3129 - ≥60 years | Income | *Taken from data table in paper*  Income *P* 0.592 (low-income class)  *P* 0.716 (middle income class) | 7/8 |
| Almedia N et al (2017) | Brazil | Data taken from previous study conducted in Cuiabá | - Total 573 - ≥60 years | Education  Income  Occupation | Education OR 0.71 SE 0.39  *Taken from data table in paper*  Income *P* 0.006  Occupation *P* 0.542 | 7/8 |
| Murphy C et al (2018) | United States | Data from Medical Expenditure Panel Survey (MEPS, 2008-2014) | - Total 24,804 - ≥18 years ≥60 years n= 13804 | Education  Employment | *Taken from data table in paper*  Education less than high school PR 1.13 (CI 1.07-1.20)  College degree or higher PR 0.98 (CI 0.92-1.04)  Employment unemployed PR 0.68 (CI 0.64-0.73) | 7/8 |
| Marques P et al (2019) | Brazil | Data from the Brazilian Older Adults Frailty (Fibra) study | - Total 2217 - ≥65 | Education  Income | Education – OR 1.32 SE 0.12  Income – OR 1.01 SE 0.10 | 7/8 |
| Silva M et al (2018) | Brazil | Survey January -February 2014 in Minas Gerais | - Total 2619 - All ages - ≥60 *n=1597* | Education | Education  OR 1.57 SE 0.08 | 6/8 |
| Morin L et al (2018) | Sweden | Cohort study registered data November 1^st^ 2010 – December 20^th^ 2013 | - Total 1,742,336 - Aged ≥65 years | Education | Education  OR 1.45 SE 0.00 | 8/8 |
| Pereira K et al (2017) | Brazil | Data collected in Florianopolis – 2009-2010 | - Total 1705 - Aged ≥60 years | Education  Income | Education  OR 1.21 SE 0.10  Income  OR 0.95 SE 0.12 | 8/8 |
| Bazargan M et al (2017) | United States | Interviews carried out 2013-2014, African Americans | - Total 400 - Aged ≥65 years | Education | Education  OR 1.55 SE 0.29 | 7/8 |
| Salih S et al (2013) | Saudi Arabia | Medical outpatients at internal medicine clinics King Abdulaziz Medical city Riyadh 1^st^ March 2009- 31 December 2009 | - Total 766 - Aged≥12   *n=413 ≥61* | Education | Education  OR 3.48 SE 0.24 | 8/8 |
| Haider S et al (2009) | Sweden | Three national registers : Swedish Prescribed Drug Register, the national inpatient register and the national Education Register | - Total 626,258 - Aged 75-89 | Education | Education OR 1.29 SE 0.01 | 8/8 |
| Chan D et al (2009) | Taiwan | Longitudinal observational study – disabled Taiwanese elderly from the ANLTCNT (Assessment of National Long-Term Care Need in Taiwan) June 2001 – June 2002 | - Total 11,788 - ≥65 years | Education | *Taken from data table in paper*  Education 1-6 years1.37  ≥7 years 1.33 | 7/8 |
| Bui D et al (2021) | Vietnam | Survey for Type 2 Diabetic patients in district hospital ( 3 districts hospitals within Quynh Phu District and Vu Thu District). | - Total 806 - Aged ≥40 *n=714 ≥55* | Education  Occupation  Wealth | Education  OR 0.73 SE 0.18  Occupation  OR 1.03 SE 0.19  Wealth  OR 1.23 SE 0.23 | 8/8 |
| Lim L et al (2017) | Malaysia | Malaysian Elders Longitudinal Research (MELoR) cohort | - Total 1256 - Aged ≥55 years | Education  Employment | Education  OR 0.75 SE 0.13  Employment  OR 1.36 SE 0.15 | 8/8 |
| Zhang X et al (2021) | China | Nationwide prospective cohort study – randomly selected from six tertiary level hospitals survey | - Total 9062 - Aged ≥65 | Education | Education  OR 0.70 SE 0.07 | 8/8 |
| Nitya S et al (2021) | India | Geriatric Health Clinics across rural health centres | - Total 207 - Aged ≥60 | Education  Social Class  Employment | Education  OR 1.23 SE 0.30  Social class  OR 1.35 SE 0.33  Employment  OR 1.75 SE 0.30 | 7/8 |
| Aljawadi M et al (2022) | Saudi Arabia | Survey using Saudi National Survey for Elderly Health (SNSEH) | - Total 2946 - Aged ≥60 | Education  Income | Education  OR 1.21 SE 0.08  Income  OR 0.94 SE 0.08 | 8/8 |
| Rezende G et al (2021) | Brazil | Survey conducted April – September 2014 | - Total 1,016 (*sample taken)* - Aged ≥60 | Education | *Taken from data table in paper*  Education *P* 0.620 | 7/8 |
| Gomes M et al (2019) | Brazil | Cross sectional study – prescriptions provided to older patients at primary health care units September 2016 – July 2017 | - Total 386 - Aged ≥60 | Education  Income  Employment | Education  OR 0.97 SE 0.26  Employment  OR 1.13 SE 0.32 | 8/8 |
| Rasu R et al (2019) | United States | Medical Expenditure Panel Survey (MEPS) 2005-2008 | - Total 4775 - Aged ≥65 | Education  Income | Education  OR 1.14 SE 0.07  Income  OR 1.24 SE 0.09 | 7/8 |
| Yang M et al (2015) | China | Data from the Comprehensive Geriatric Assessment and Health care Service System in Chinese Elderly Project | - Total 717 - Aged ≥60 | Education | Education  OR 0.59 SE 0.22 | 7/8 |
